# Echidna Venom Gland Transcriptome Provides Insights into the Evolution of Monotreme Venom

**DOI:** 10.1371/journal.pone.0079092

**Published:** 2013-11-12

**Authors:** Emily S. W. Wong, Stewart Nicol, Wesley C. Warren, Katherine Belov

**Affiliations:** 1 Institute for Molecular Bioscience, University of Queensland, QLD, Australia; 2 School of Zoology, University of Tasmania, TAS, Australia; 3 The Genome Institute, Washington University School of Medicine, St. Louis, Missouri, United States of America; 4 Faculty of Veterinary Science, The University of Sydney, NSW, Australia; Fordham University, United States of America

## Abstract

Monotremes (echidna and platypus) are egg-laying mammals. One of their most unique characteristic is that males have venom/crural glands that are seasonally active. Male platypuses produce venom during the breeding season, delivered via spurs, to aid in competition against other males. Echidnas are not able to erect their spurs, but a milky secretion is produced by the gland during the breeding season. The function and molecular composition of echidna venom is as yet unknown. Hence, we compared the deeply sequenced transcriptome of an in-season echidna crural gland to that of a platypus and searched for putative venom genes to provide clues into the function of echidna venom and the evolutionary history of monotreme venom. We found that the echidna venom gland transcriptome was markedly different from the platypus with no correlation between the top 50 most highly expressed genes. Four peptides found in the venom of the platypus were detected in the echidna transcriptome. However, these genes were not highly expressed in echidna, suggesting that they are the remnants of the evolutionary history of the ancestral venom gland. Gene ontology terms associated with the top 100 most highly expressed genes in echidna, showed functional terms associated with steroidal and fatty acid production, suggesting that echidna “venom” may play a role in scent communication during the breeding season. The loss of the ability to erect the spur and other unknown evolutionary forces acting in the echidna lineage resulted in the gradual decay of venom components and the evolution of a new role for the crural gland.

## Introduction

**Figure 1:**
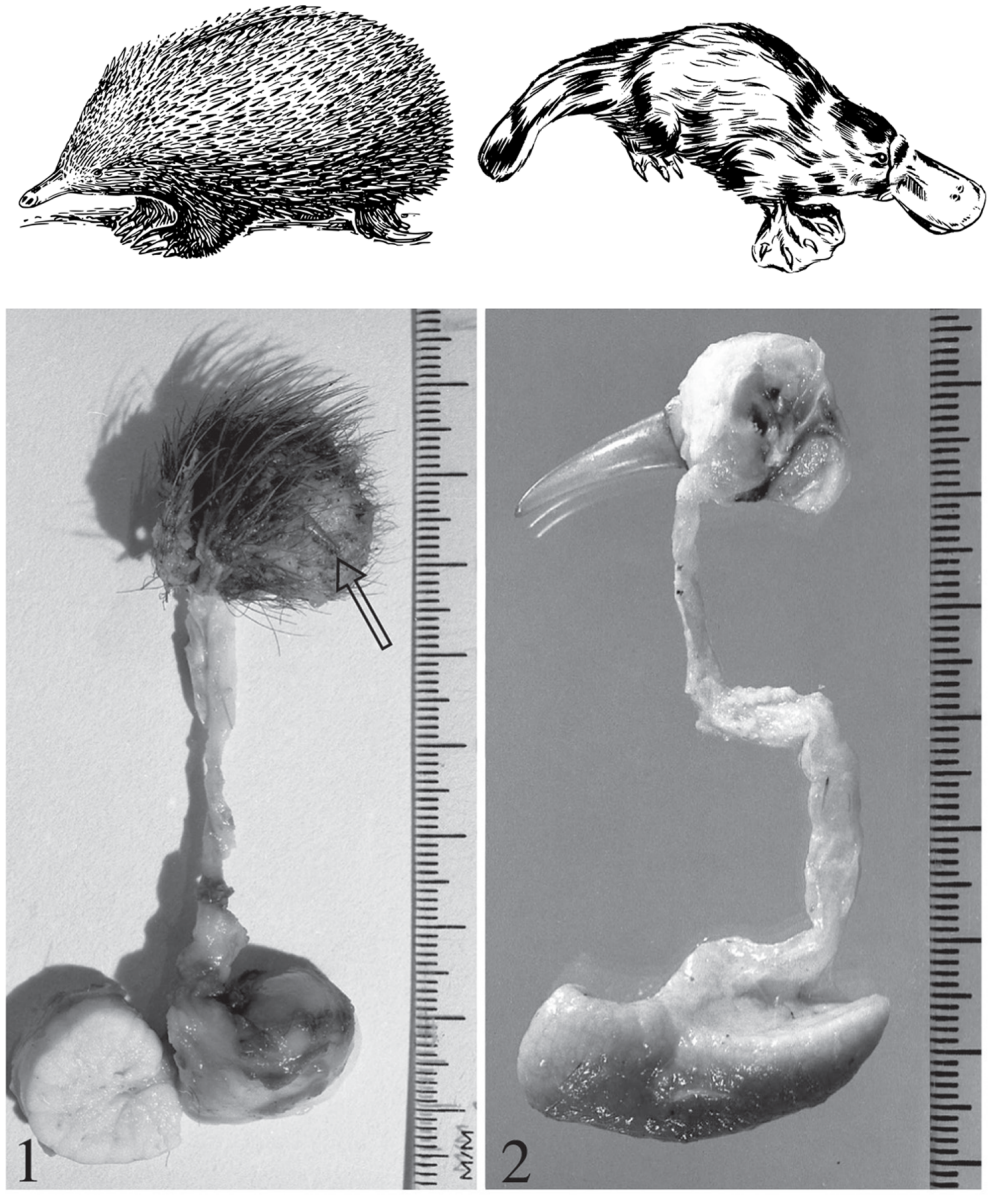
Monotremes and their crural glands. 1) An echidna (image taken from http://faunafemales.wikispaces.com/Echidna) and a dissected crural gland-spur apparatus from an adult male echidna. Photo was taken by William Krause and reprinted with permission from reference 2. Note that the main duct linking the crural gland and spur is shorter in the echidna than in the platypus. The spur (highlighted by an arrow) is not able to be erected in the echidna. 2) A platypus (image taken from http://faunafemales.wikispaces.com/Platypuses) and a dissected gland-spur apparatus taken from an adult male platypus. The original photograph was published by Krause in reference 2 (licence number 3184480567659).

Monotremes are egg-laying mammals and include the platypus (*Ornithorhynchus anatinus*), the short-beaked echidna (*Tachyglossus aculeatus*) (shown in Figure 1), and the long-beaked echidnas (*Zaglossus* sp). It has been long recognised that male platypuses have spurs on their hind legs that are connected to venom glands by venom ducts. The venom glands increase in size during the breeding season [1], [2]. During this period, males exhibit aggressive behaviours towards other males. In the platypus the venom is delivered into its victim through spurring events. The venom contains a complex mixture of peptides that cause intense pain and swelling. The recent sequencing of the platypus genome and the venom transcriptome has allowed us to more comprehensively characterise many of these peptides and determine their evolutionary origins. For instance, we showed that the key component of platypus venom, the defensin-like peptides (DLPs) evolved by gene duplication from the antimicrobial peptide β-defensin genes [3]. The function of DLPs is unknown, although they do not have any antimicrobial characteristics [4]. Other platypus venom components include C-type natriuretic peptides, which is known to cause histamine release [5], and calcium influx [6], as well as hyaluronidase, amide oxidase, protease inhibitor, proteins associated with mammalian stress response pathway and immune molecules, discovered by shot-gun proteomics [7].

Male echidnas also have spurs on their hind limbs. As in the platypus, the spur is attached to a gland, however, this spur is unable to be erected and used for spurring (Figure 1). The first description of the echidna crural gland [8] described the gland in its inactive phase. However, in 1968 Griffiths [9] noted that the gland increased in size during the breeding season. This gland produced a milky substance which was secreted during the breeding season. However, there have been no accounts of echidna envenomations and it is not known whether the secretions produced by this gland are venomous. Krause recently provided a detailed anatomical description of the echidna crural gland and confirmed cyclic activity of the gland [2]. He showed an increase in secretory granules in the secretory epithelium of the gland during the breeding season. In the platypus, exocytosis of this secretory epithelium was observed. However in the echidna, exocytosis was not observed. This suggests that there may be key differences in the function of the gland between the two species.

There is physiological, molecular and fossil evidence to suggest that the ancestor of platypus and echidnas was venomous. Firstly, both animals possess spurs, apparatuses which were likely developed to pierce the surface of the skin to inject toxic substances. Additionally, using a Bayesian molecular clock dating strategy, we have found that key toxin genes in the platypus evolved through gene duplication prior to the divergence of echidna and platypus lineages [3], which occurred in the early Cretaceous [10]. Monotremes possess a supernumerary bone, the os calcaris, which forms a plate-like bony base of the spur, and multituberculate mammals from the late Cretaceous possess an os calcaris which appears to be homologous to the monotreme structure [11]. Hurum et al argue from this that ancestral mammals were probably venomous, and that the venom gland apparatus of modern monotremes is a plesiomorphic character which has been lost in therian mammals.

To determine whether the echidna crural gland retains its ancestral venomous role, we compared an echidna in-season venom transcriptome with a platypus in-season venom transcriptome. We expected to see high levels of similarity between the venom gene repertoires of the two species. However, we found this not to be the case as platypus venom genes were not highly expressed in the echidna transcriptome.

## Methods

### Sequence Analysis

**Figure 2:**
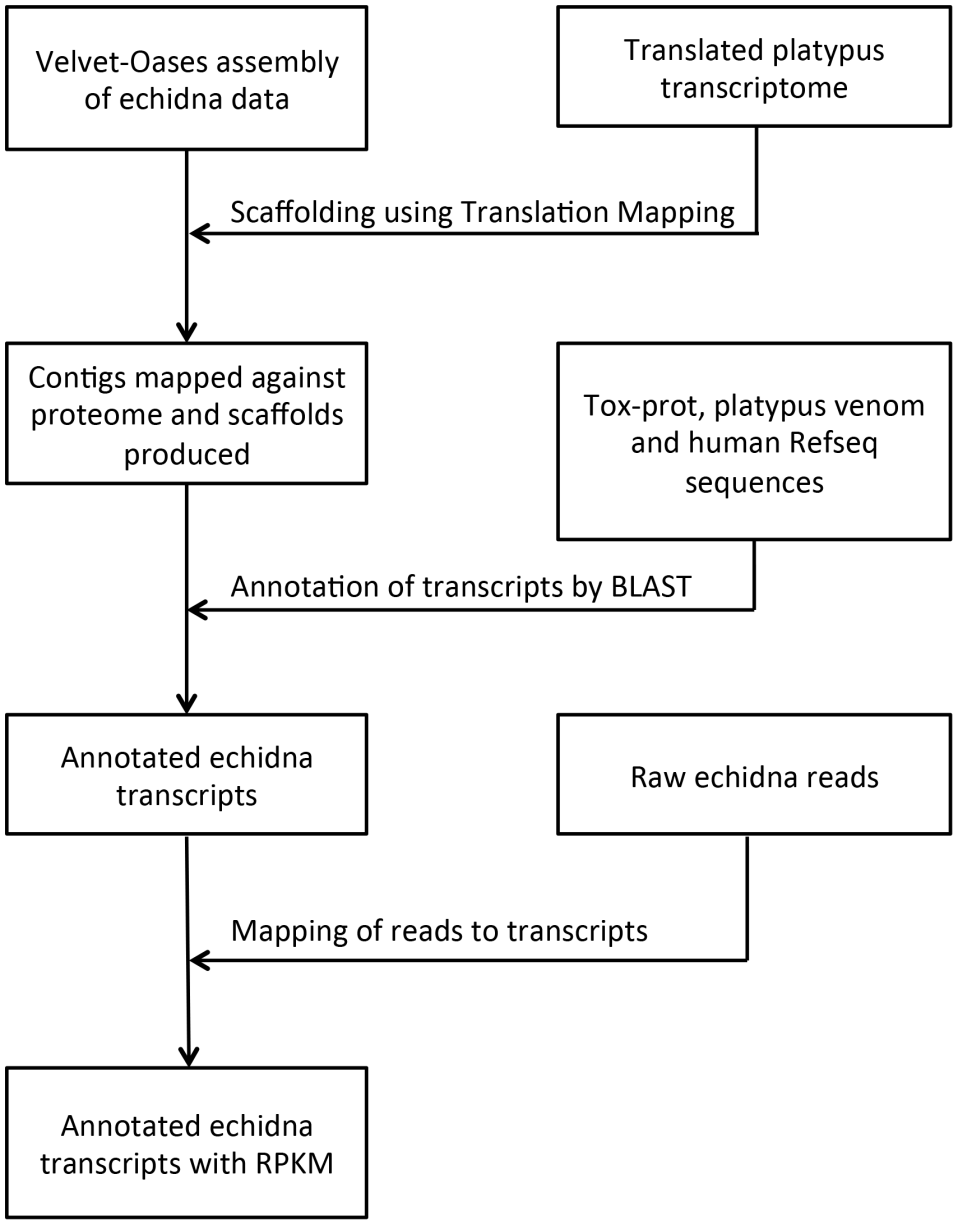
Schematic of bioinformatics workflow for the assembly, annotation and quantitation of echidna transcripts.

A schematic of the bioinformatics workflow is presented in Figure 2. A *Tachyglossus aculeatus* venom gland transcriptome was sequenced on an Illumina GAIIx instrument using previously describe methods for sample preparation and sequencing [7]. The venom gland was kindly provided by Frank Grutzner under University of Adelaide Animal Ethics Committee project number S-032-2008. The sequence reads have been deposited under accession number SRP027593 in the SRA database at NCBI. Quality filtered reads were assembled with the Velvet-Oases pipeline (kmer length = 31bp and –ins_length = 260) [12], [13]. To improve the transcriptome assembly, the Scaffolding using Translation Mapping (STM) strategy [14] was used to scaffold Oases contigs using Ensembl predicted proteins, an experimentally derived dataset of platypus venom proteins [5], [7], [15], and all human Refseq proteins as references. Data available from the Dryad Digital Repository: http://doi.org/10.5061/dryad.4qq0v. BLAST [16] searches were performed against a database containing all Tox-Prot proteins [17], additional platypus venom toxins that have not been yet included in the Tox-Prot database [7] and human Refseq sequences using scaffolds derived from STM to identify possible homologies between echidna and other venom toxin proteins.

### Expression Quantification and Comparison

Expression levels for each scaffold were derived by first mapping the raw sequencing reads to transcript scaffolds using Bowtie [18]. We then generated digital counts for each scaffold by counting reads that map back to transcripts. Multi-mapping reads were discarded. To compare the most highly expressed proteins between echidna and platypus venom, we ranked the expression values (normalized using Reads Per Kilobase per Million (RPKM) [19]) of the echidna transcripts, from highest to lowest, and compared it with similarly ranked list from a platypus transcriptomic dataset described in [20]. Echidna transcripts were annotated with respect to both human and platypus genes. The top 100 genes from both lists were compared and functional enrichment analysis was performed using Ontologizer [21] with orthologous human gene names as input. GoStat2 [22] was used to compare overrepresented ontology terms from highly expressed echidna genes versus the same in platypus. False discovery rate (FDR) [23] corrections were used for all functional term enrichment analyses. Ranked gene lists for comparisons only included genes that map to a homologous protein and did not include unnamed genes. If multiple scaffolds map to the same gene name, the name is only included once in the list.

## Results and Discussion

The Oases assembly comprised of 72,549,524 base pairs with an average transcript length of 751 base pairs, a maximum transcript length of 25,037 base pairs and an N50 of 1,474 bases. To improve our assembly we mapped these contigs against the platypus proteome to scaffold contigs aligning to the same protein in the proteome. The platypus proteome was chosen because it is the most closely related species with a sequenced genome. Moreover, it facilitates the identification of the echidna homologs of platypus venom components that have been conserved between the two species. Our reference proteomes contain both Ensembl predicted proteins, derived from the translation of mRNAs of predicted genes in the platypus genome, as well as proteomic data that was generated using shot-gun proteomics [7].

### Tox-Prot Comparison

We compared our scaffolds to sequences in a toxin database, Tox-Prot, to identify putative venom proteins based on homology. As venom proteins have typically evolved from body proteins, to distinguish between venom proteins and related body proteins we also searched against the human Refseq sequences. Sequences were considered as possible toxins if they were more similar to known toxins than a homologous protein from human. 30 transcripts from 15 genetic regions matched 12 unique toxin sequences with a lower BLAST e-value than to a human protein (Table 1). Five transcripts of proteins found in platypus venom were identified: *CD55*, amide oxidase, corticotrophin-releasing factor binding protein, a Kunitz domain-containing protease inhibitor and a cysteine-rich secretory protein (Table 2). Echidna sequences also shared similarity with a subunit stonefish toxin with haemolytic activities [24], snake nerve growth factors, protease inhibitors from snake and sea anemone venom with ion channel inhibitory activity (sea anemone) [25], and myotoxic and neurotoxic snake phospholipase A2s [26], [27].

**Table 1:** Echidna transcripts that display higher similarity to known toxins than human proteins.

Toxin name	Transcript locus number	Expression rank (out of 91,300)
Stonustoxin subunit alpha (Acc: Q98989)	726, 11716	24,455
Platypus CD55/complement decay accelerating factor (Acc: K4PB89)	2792, 50224	42,570
Platypus amide oxidase (Acc: K4P911)	8792, 18049	71,866
Snake venom nerve growth factor 2 (*Tropidechis carinatus*) (Acc: Q3HXX7)	11196	36,479
Snake venom nerve growth factor (*Naja naja*)(Acc: P01140)	11197	20,498
Platypus corticotrophin-releasing factor binding protein (Acc: K4PLU9)	11716	79,938
Platypus Kunitz domain containing protease inhibitor	16527	61,251
Sea anemone Kunitz domain containing protease inhibitor (Acc: Q9TWG0)	16966	41,527
Snake mulgin-3 protease inhibitor (*Pseudechis australis*) (Acc: Q6ITB9)	16966, 47678	35,771
Platypus cysteine-rich secretory protein (Acc: K4P991)	40579	51,113
Snake venom phospholipase A2 (*Echis carinatus*)(Acc: P48650)	43122	53,680
Snake venom phospholipase A2 trimucrotoxin (*Protobothrops mucrosquamatus* ) (Acc: Q90W39)	49881	83,368

The name of the known toxin together with the echidna transcript number and ranking of mRNA expression level are shown (a higher ranking indicates higher expression levels relative to other transcripts in the echidna transcriptome).

**Table 2:** Known platypus venom proteins and their homologs in the echidna transcriptome.

Platypus toxin	Ancestral activity	Derived activity	Expressed in echidna gland
Defensin-likepeptides	Antimicrobial	Unknown	No
Nerve growthfactor	Regulation of nerve growth and differentiation; Hyperalgaesia	Possibly hyperalgaesia	No
C-type natriureticpeptide-like	Natriuretic, diuretic and vasodilatory functions associatedwith homeostasis and blood pressure control andregulates the growth and differentiation ofcartilaginous growth plate chondrocytes	Relaxant, oedema-producing andmast-cell degranulatingactivities	No
Hyaluonidase	Increase tissue permeability by lowering the viscosity of hyaluronan– roles in reproduction	Believed to accelerate the spread oftoxins and hemostatic factorsin toxin	No
Amide oxidase	Function in cell differentiation, growth, wound healing,detoxification, cell signaling	May cause platelet aggregation,assist in haemorrhage andcell death	Yes
Peptidoglycanrecognitionprotein-1	Antimicrobial	Unknown	No
Serpin	Blood coagulation, complement activation, fibrinolysis,angiogenesis, inflammation, tumour suppression andhormone transport	Possible role in blood coagulationand hypertension	No
Kunitz-domaincontaining serineprotease inhibitor	Involved in hemostasis	Possible role in disrupting hemostasis	Yes
Nucleobindin	Stimulates autonomic nervous system activity, increasesblood pressure, induces fear in rats	May induce the fear response in victim	No
Differentiationfactor-15	Regulates the inflammatory response and apoptosis	May function to induce pain	No
CXC-chemokine	Chemotactic, mediate cell growth and triggers aninflammatory response	Unknown	No
Complement decay-acceleration factor	Inhibits complement-mediated lysis	Unknown	Yes
L-D-amino-acid-residue isomerase	Unknown	Interconverts the second amino-acidresidue of venom peptides betweenthe L-form and D-form	No
Corticotropin-releasingfactor-binding protein	Linked to behavioural and psychological changes andnerve signalling	Suggested to increase submissive behaviourin victim linked to role of venomfor intraspecific competition	Yes

We further examined the expression levels of these echidna transcripts to gain insights into their putative function. We found that all identified echidna protein-coding transcripts with homology to known toxins were lowly expressed in the transcriptome (Table 2). Pharmacologically characterized venom proteins have been found to be some of the most highly expressed genes in the platypus venom gland transcriptome [7], [20]. Using this logic the low expression of echidna toxin transcripts suggests that venom production is not a key role of this gland.

### Analysis of most Highly Expressed Genes

To provide insight into the function of echidna venom, we examine the most highly expressed genes in the echidna venom gland. Quantification of expression levels involved the counting of raw reads that map to assembled scaffolds. The numbers were normalized with respect to the length of the scaffolds. This method allowed us to examine the most highly expressed genes and allowed comparison with similar experiments in platypus. None of the top 50 most highly expressed echidna genes were found in the top 50 of platypus. Similar results were obtained when human protein-coding transcripts instead of platypus ones were used to assemble the contigs. Comparison of the top 200 most abundant transcripts from the two datasets identified three proteins common to both echidna and platypus glands: a mitochondrial phosphate carrier protein (*SLC25A3*), mesencephalic astrocyte-derived neurotrophic factor (*MANF*) and a ribosomal protein (*RPL9*). The most highly expressed genes in the echidna venom gland are shown in Table 2. The most abundant venom components in platypus venom, venom defensin-like peptides and C-type natriuretic peptides [20], were not identified in echidna (Table 3).

**Table 3:** The most highly expressed proteins in the echidna transcriptome as annotated by platypus Ensembl proteins.

Transcript reference number	Platypus Ensembl accession	Gene name	Description	RPKM
968	ENSOANG00000011369	BCHE	butyrylcholinesterase	604
8337	ENSOANG00000005594	SLC25A3	solute carrier family 25 (mitochondrial carrier)	441
1723	ENSOANG00000009799	CALR	calreticulin	433
15082	ENSOANG00000012964	EEF1B2	eukaryotic translation elongation factor 1 beta 2	421
1219	ENSOANG00000013689	–	–	400
1766	ENSOANG00000003422	LTV1	unknown function	378
656	ENSOANG00000011189	RPS27A	ribosomal protein S27a	376
6048	ENSOANG00000000439	–	–	371
3603	ENSOANG00000010996	PDCD10	programmed cell death 10	367
871	ENSOANG00000008043	–	–	366

### Gene Ontology Analysis

We compared gene ontology (GO) enrichment statistics between the expressed genes (RPKM>10) in the echidna and platypus datasets to examine and infer biological differences between the two species by restricting the analysis to the top 200 most highly expressed genes in both datasets. 182 out of the top 200 echidna genes were associated with 1,537 GO annotations that represented 580 unique terms. Compared to the platypus dataset, we detected five significantly enriched GO terms that included adenyl ribonucleotide binding, adenyl nucleotide binding, ATP binding, post-translational modification and plasma membrane (Table 4).

**Table 4:** Gene ontology (GO) terms that were enriched in the top 200 most highly expressed echidna genes compared to the platypus using GoStat2.

GOterm	GOname	Echidna genesin group	Adjustedp-value
GO:0032559	adenylribonucleotide binding	chuk; supv3l1; lars; igf1r; helz; tarsl2; tpr; atp2c1; dhx29; wee1;crebbp; smarca1; dhx36; dync1h1; hspa4; smchd1;dync1li1; tcn1; myo10; smarca5; taok1; atp2a2;abl1; ndufa10; trio; atp2b1; ddx50; top2b; afg3l2;hnrnpu; kif5b; ddx6	0.0159
GO:0030554	adenylnucleotide binding	chuk; supv3l1; lars; igf1r; helz; tarsl2; tpr; atp2c1;dhx29; wee1; crebbp; smarca1; dhx36; dync1h1;hspa4; smchd1; dync1li1; tcn1; myo10; smarca5;taok1; atp2a2; abl1; ndufa10; trio; atp2b1; ddx50;top2b; afg3l2; hnrnpu; kif5b; ddx6	0.0159
GO:0005524	ATPbinding	chuk; supv3l1; lars; igf1r; helz; tarsl2; tpr; atp2c1;dhx29; wee1; crebbp; smarca1; dhx36; dync1h1;hspa4; smchd1; dync1li1; tcn1; myo10;smarca5; taok1; atp2a2; abl1; ndufa10; trio;atp2b1; ddx50; top2b; afg3l2; hnrnpu; kif5b; ddx6	0.0159
GO:0043687	post-translationalprotein modification	chuk; mgrn1; cnot4; abl1; usp14; igf1r; trio; ppp2ca;dsp; rb1cc1; twf1; ube2d2; prdx4; march7; wee1;wwp2; crebbp; cul4b; ighm; huwe1; ppp1cc; ppp3ca;ccdc88c; march6; taok1	0.0257
GO:0044459	plasmamembrane part	clcn4; atp2a2; igf1r; slc12a7; mpp1; cltc; dsp;rab5a; cyfip1; enpp3; slc4a4; atp2b1; dtna;apc; calr; cp; cdh1; ighm; tjp1; itgb1; slc7a8; slc25a3	0.0843

Threshold at adjusted p-value<0.1.

We also performed functional term analyses on the top 200 most highly expressed genes in both echidna and platypus datasets. No gene ontology terms were found to be significant at p<0.05 after multiple testing correction (FDR) in independent tests for each species.

These analyses confirm that the key role of the echidna crural gland is not venom production. The life history of echidnas may point to the likely functional role of this gland. Echidnas are usually solitary, but during the breeding season (June to September) they are believed to use chemical cues to attract mates [1], [28]. Both sexes exude a pungent, musky odour possibly from the cloaca to advertise their reproductive status [29]. Male echidnas also exude a white milky substance from the base of their non-erectable spurs during the breeding season, while females, who lose their spurs before maturity, produce a small amount of solid secretion from the pits previously occupied by the spurs [28]. Several authors have speculated that the primary function of this white exudates acts as a chemical attractant during mating [2], [28].

The recent study by Harris *et al*. [28] investigated the composition of waxy secretions from the base of the spur using a mass spectrometry strategy to identify non-peptide compounds. The study revealed a variety of large molecules in produced by the tissue around the spur collar including sterols, fatty acids and methyl esters supporting their function in some form of scent communication. Consistent with this finding, stearoyl-CoA desaturase (*SCD*) – a protein involved in fatty acid biosynthesis was highly expressed in the echidna transcriptome. Other proteins involved in fatty acid metabolism including acyl-CoA dehydrogenase (*ACADVL*), acetyl-CoA carboxylase beta (*ACACB*), acyl-CoA synthetase long-chain family member 1 (*ACSL1*) as well as lecithin-cholesterol acetyltransferase (*LCAT*), sortilin-related receptor, L(DLR class) Repeats-containing protein (*SORL1*) and the Niemann-Pick disease protein (*NPC1*) are also all involved in sterol metabolic processes and expressed in the echidna crural gland transcriptome. Similar sterols and fatty acids are commonly found in scent gland secretions from other animals and can function to prolong the life of a scent mark (sterol esters) and encode complex informational content (long-chain fatty acids) (reviewed in [28]).

The major components of secretory granules in echidna crural glands are proteins [2] and it may be that the spur secretions possess additional, as yet, unknown roles. The most highly expressed gene in the echidna crural gland is most similar to butyrylcholinesterase, a serine hydrolase, whose physiological function in humans is still unclear, although it has been found to be an effective detoxification enzyme [30], [31]. Further research involving functional assays is required to determine the true biological roles of the most highly expressed proteins in echidna spur secretions.

Our results suggest that the primary role of the echidna crural gland is not to produce venom for offensive or defensive purposes. The majority of known platypus toxins were not detected in the transcriptome of the echidna crural gland. 30 echidna scaffolds matched 12 known toxins from a number of venomous species, but these were only lowly expressed in the gland and perhaps provide a remnant of the glands life history. Today the transcriptomes of the echidna and platypus crural glands have distinct expression profiles sharing few similarities in their most highly expressed genes. Should the echidna genome become available a review of the evolutionary remnants of venom genes can be conducted. The echidna gland appears to have evolved to play a role in chemical communication during the breeding season. Although the function of the crural gland has diverged significantly since the last common monotreme ancestor, each lineage continues to use the gland to aid reproduction.
